# Updating statistical practice in ecotoxicology: reflections and recommendations

**DOI:** 10.1093/inteam/vjaf029

**Published:** 2025-04-30

**Authors:** S Jannicke Moe, David R Fox, Raoul Wolf

**Affiliations:** Norwegian Institute for Water Research (NIVA), Økernveien 94, 0579 Oslo, Norway; Senior Editor, Integrated Environmental Assessment and Management; Environmetrics Australia, Beaumaris, Victoria, 3193, Australia; Norwegian Geotechnical Institute (NGI), Sandakerveien 40, 0484 Oslo, Norway

Approaches to statistical analysis of data from ecotoxicity testing emerged in the 1980s and have grown over the decades, but with only intermittent involvement of statisticians. Consequently, statistical practices in this field have evolved in various directions. Fragmented, inconsistent, and outdated use of statistical methods for ecotoxicology by authorities and jurisdictions have further contributed to a landscape which can be confusing to navigate. A prominent example is the issue of whether no-observed effect concentrations (NOECs) should be banned from or used in regulatory ecotoxicology, which has been debated for more than 30 years (e.g., Laskowski, 1995; van Dam et al., 2012).

Other scientific disciplines, e.g., ecology, psychology, and medicine, have refined and optimized their statistical toolboxes in closer collaboration with statisticians in recent decades. While ecotoxicology has benefitted from the progress of these related disciplines, regulatory risk assessments are still largely based on statistical principles and approaches that can no longer be considered state-of-the-art.

Within the Society for Environmental Toxicology and Chemistry (SETAC), recent initiatives related to statistics with regulatory relevance have experienced a high level of interest and discussion. Activities initiated in 2024 include the proposal to establish a SETAC interest group for statistics and the launch of a special series on statistical methods in *Integrated Environmental Assessment and Management*. Moreover, many ecotoxicologists have expressed interest in the revision of OECD document no. 54, “Current approaches in the statistical analysis of ecotoxicity data: a guidance to application” (OECD, 2006). The document is no longer considered reflective of contemporary statistical methods or computational platforms available to ecotoxicology, and a revision is planned for 2026. The German Environment Agency (UBA) will coordinate scientific contributions to the revision process (Daniels et al., 2024), and organized the “1st UBA expert workshop on the OECD No. 54 revision” in September 2024 focusing on three chapters from OECD document no. 54: chapter 4, “Hypothesis testing,” chapter 5, “Dose-response models,” and chapter 6, “Biological effect models.” While a summary from the workshop by the organizers can be expected at a later point, we will here share some reflections on chapters 4 and 5.

In its current version, OECD document no. 54 proposes a distinction between “hypothesis testing” (ANOVA-type models) and “dose-response modeling” (regression models). This dichotomy neglects that common statistical concepts are underlying both approaches. Both ANOVA-type models and regressions are variants of linear models in statistics. Both involve estimation of parameters, and both use likelihood or information criteria to compare hypotheses. The main difference in this context is that “hypothesis testing” treats concentrations as categories, while “dose-response models” use the concentration as a continuous predictor variable to estimate parameters of a regression equation.

Ecotoxicologists now have access to a wide variety of statistical tools for combined hypothesis testing and dose-response modeling via generalized linear models (GLMs) as well as more advanced and flexible nonlinear models. Basic GLMs can make of use link functions (generalization) instead of data transformation to mimic normal-based models, for example, for logistic regression. Hierarchical versions (mixed-effect GLMs) can better capture nested structures and variability in data (Green, 2015). Furthermore, generalized additive models (GAMs) allow description of relationships between predictor variables and the response by smooth curves (Pedersen et al., 2019); these can therefore be powerful tools for exploring nonlinear patterns in dose-response data. Open-source statistical software, such as R (R Core Team, 2024), has made these methodologies and frameworks readily available to scientists and regulators worldwide.

Dose-response curves can be fitted with a range of models containing 2–5 parameters (Ritz et al., 2015), for obtaining parameter values or derived values such as the ECx (concentration associated with x% effect). Alternative metrics such as the benchmark dose (BMD; EFSA Scientific Committee et al., 2022) and the no-significant effect concentration (NSEC; Fisher & Fox, 2023) have been proposed more recently. These alternatives have different properties, e.g., regarding sensitivity to low sample size, computational complexity, and suitability as input to higher-level assessment such as species sensitivity distributions. Further exploration and comparison of these methods can help us gain more insight into these alternatives, and, for example, establish criteria for recommending one method over another.

Our reflections can be concluded with a “wish list” for the OECD document No. 54 revision process:

- Clarify the connections between hypothesis testing (chapter 4) and dose-response models (chapter 5). Let continuous regression-based models be the default choice whenever possible and ANOVA-type models be the special case when strictly necessary.
- Include contemporary and well-established statistical tools, e.g., GLMs, in the recommended toolbox for ecotoxicologists, ideally supported with references to well-established statistical software, packages, and online platforms.
- Present Bayesian methods as an alternative to their frequentist counterparts; both frameworks come with strengths and weaknesses, and both should adhere to well-established best practices.

In the longer term and more generally, we would welcome these developments:

- Stronger collaboration with regulatory authorities and jurisdictions for better clarification of issues such objectives of ecotoxicity testing, statistical design considerations, recommendations on modes of analysis, and terminology.
- Higher awareness regarding the importance of statistical design and methodology for reducing animal testing in ecotoxicology.
- More investment in training in statistical science and data literacy for ecotoxicologists, beyond sporadic courses and specialized fields of research within ecotoxicology. This can include topics such as experimental design, visual inspection, best practices for all types of regression models, and knowledge of Bayesian frameworks.
- Better integration of process-based and statistical models for obtaining toxicity estimates.

In moving forward, progress should integrate learning from the past decades (Chapman et al., 1996). Improved statistical practices for regulatory ecotoxicology can support a better assessment and decision-making by authorities regarding chemical hazard and risk. With significant interest from industry, academia, and regulators aligning in recent years, the time seems ripe for an overhaul.
